# The Precision Paradox in Prostate Cancer Diagnostics: Grade Migration, Risk Misclassification, and Overtreatment in the mpMRI-Targeted Biopsy Era

**DOI:** 10.3390/cancers18111700

**Published:** 2026-05-23

**Authors:** Andrea Micillo, Simone Steffani, Luca Orecchia, Roberto Miano, Eric Walser, Guglielmo Manenti

**Affiliations:** 1 Diagnostic Imaging Unit, Casa di Cura Villa Delle Querce, 00040 Nemi, Italy; 2 Diagnostic Imaging Department, AOU Policlinico Tor Vergata, 00133 Rome, Italy; 3 Department of Surgical Sciences, University of Rome Tor Vergata, 00133 Rome, Italy; 4 Urology Unit, AOU Policlinico Tor Vergata, 00133 Rome, Italy; 5 Sperling Prostate Center, Delray Beach, FL 33445, USA

**Keywords:** prostate cancer, mpMRI, targeted biopsy, precision paradox, grade migration, overtreatment, focal therapy

## Abstract

The diagnostic field of prostate cancer (PCa) has undergone a significant evolution with the widespread integration of multiparametric magnetic resonance imaging (mpMRI) and mpMRI-targeted biopsies (TBx). This approach has been shown to improve the detection of clinically significant prostate cancer (csPCa) while reducing the overdiagnosis of low-risk disease. However, a conceptual and clinical challenge, which can be referred to as the “Precision Paradox,” has emerged. By directing biopsy cores almost exclusively into the most suspicious MRI lesions, clinicians may inadvertently overrepresent the biological significance of a limited high-grade component. This can lead to grade migration and pathological downgrading at the time of radical prostatectomy (RP). Although downgrading does not automatically equate to clinical overtreatment, it introduces prognostic uncertainty that complicates risk stratification for active surveillance (AS) and focal therapy. This conceptual commentary provides a critical perspective on this diagnostic issue. We synthesize recent meta-analyses to evaluate the true rates of grade mismatch associated with TBx and combined biopsy approaches. Furthermore, we discuss the spatial limitations of biopsy sampling, the pathological mechanisms driving grade discordance, and the clinical relevance of minor high-grade components such as cribriform architecture. Finally, we highlight the role of multi-omics and validated genomic biomarkers in risk models, ultimately fostering improved shared decision-making in the modern mpMRI era.

## Simple Summary

Modern prostate cancer (PCa) diagnosis increasingly relies on multiparametric magnetic resonance imaging (mpMRI) and targeted biopsy (TBx) to sample the most suspicious areas of the prostate gland. While this has been shown to improve the detection of aggressive disease and minimize the diagnosis of indolent tumors, it can also introduce a bidirectional diagnostic tension. Sampling only the most abnormal focal spot may occasionally make cancer appear globally more severe on biopsy than it truly is across the entire prostate, a phenomenon that could be called the “Precision Paradox.” Consequently, some patients may experience pathological downgrading at the time of radical prostatectomy. This conceptual commentary discusses why this discordance occurs, analyzing the differences between biopsy and surgical grading rules, the spatial limitations of both systematic and targeted sampling, and the impact of tumor volume. Furthermore, we explore the integration of multi-omics, tissue biomarkers, and enhanced patient care to ensure that treatment decisions accurately match the biological reality of the disease, balancing the risks of undertreatment and overtreatment.

## 1. Introduction

The diagnostic pathway for prostate cancer (PCa) has been fundamentally transformed by the introduction of multiparametric magnetic resonance imaging (mpMRI) and standardized reporting systems such as the Prostate Imaging Reporting and Data System (PI-RADS) [1,2]. These advancements now guide transrectal ultrasound (TRUS) or transperineal fusion platforms to perform targeted biopsies (TBx) [3,4,5]. Current European Association of Urology (EAU) guidelines recommend performing mpMRI prior to biopsy, followed by targeted and regional or systematic sampling to maximize the detection of clinically significant prostate cancer (csPCa) [6].

However, this transition has introduced a complex clinical phenomenon which could be referred to as the “Precision Paradox” [4,7]. In this context, the paradox refers to the opposing dynamic of highly accurate focal sampling of the most aggressive tumor component and the potential misrepresentation of the whole-gland tumor burden [8,9]. When clinical practice relies primarily on the cores taken from the most suspicious MRI lesions, there is a risk of overestimating the overall tumor grade compared to the final whole-gland histology [9,10,11]. This creates “grade migration,” which can theoretically expose patients to overtreatment, subjecting them to radical whole-gland therapies for a disease burden that might have been safely managed with organ-sparing strategies such as active surveillance (AS) or Focal Therapy (FT) [2,4,12,13,14].

This article serves as a conceptual commentary to critically evaluate this diagnostic challenge. Rather than presenting TBx as inherently flawed, we aim to provide a balanced perspective that explores both the risks of focal overestimation and the persistent dangers of undersampling. By evaluating recent large-scale meta-analyses, pathological grading mechanisms, and the integration of novel multi-omics and genomic biomarkers, we seek to contextualize the precision paradox and offer nuanced strategies for modern PCa management.

## 2. The Bidirectional Diagnostic Tension: Overestimation vs. Undersampling

To properly contextualize the Precision Paradox, it is advised to acknowledge the bidirectional nature of PCa diagnostics [6,15]. The integration of mpMRI and TBx provides meaningful clinical value; it effectively reduces the diagnosis of indolent, low-risk PCa and improves the detection of csPCa compared to systematic biopsy (SBx) alone [3,15,16,17]. In a screening or diagnostic setting, mpMRI serves as an invaluable triage test, allowing many men with negative imaging to safely avoid unnecessary biopsies [6,12,18,19,20,21].

**Figure 1:**
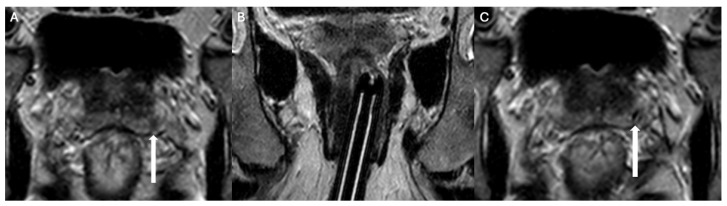
Direct MRI-Guided Targeted Biopsy Procedure of a PI-RADS 4 Index Lesion. (**A**) Axial T2-weighted MRI: index lesion in the mid-glandular left peripheral zone (arrow). (**B**) Coronal T2-weighted sequence: transrectal probe aligned toward the index lesion. (**C**) Intraprocedural axial T2-weighted image: focal gadolinium hypointensity at the needle tip confirms correct intralesional placement (arrow).

However, the precision of TBx may introduce a delicate diagnostic tension [1,22]. On one end of the spectrum is “over-precision,” where the biopsy needle directly targets the absolute peak of cellular atypia within an index lesion [4,23] (Figure 1). Because traditional risk models often dictate that the highest single biopsy grade determines the clinical risk group, a microscopic focus of high-grade disease (e.g., a tiny fraction of Gleason pattern 4) can disproportionately inflate the patient’s global risk profile [12,24,25,26].

**Figure 2:**
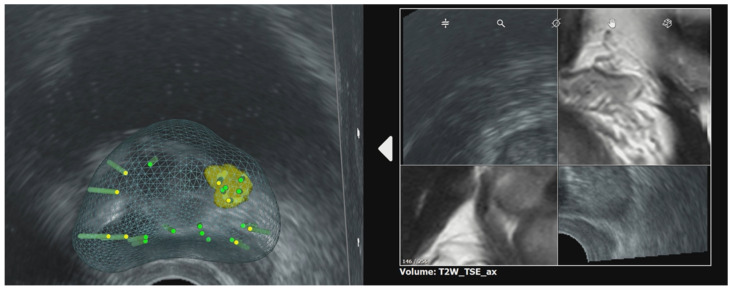
Post-procedural report from MRI/TRUS software-fusion transperineal prostate biopsy. Platform reconstruction shows the prostate volume, MRI-defined index lesion (yellow), needle trajectories (green), and orthogonal fused imaging planes. This illustrates the targeting margin and potential spatial discrepancy between the MRI-defined ROI and actual sampled tissue, crucial for interpreting perilesional cancer detection.

On the opposite end of the spectrum lies “under-precision” or undersampling [24,27]. The precision paradox is not a one-directional mechanism leading solely to overtreatment [4,8]. Studies have shown that clinically significant cancer may extend beyond the boundaries of the MRI-defined region of interest (ROI) [28,29,30]. Research evaluating the perilesional “penumbra” has demonstrated that a notable fraction of csPCa cores are located outside the primary MRI target, with up to 18% of significant cancers diagnosed exclusively in the surrounding tissue [28,31]. These findings should be interpreted with due consideration of the potential error margin inherent to fusion biopsy (Figure 2), and the role of perilesional sampling should ideally be evaluated using a more unequivocal biopsy technique, such as direct MRI-guided biopsy. Nevertheless, this suggests that TBx also suffers from severe spatial resolution limitations, and targeting too narrowly can miss clinically relevant disease extensions [8,28,31,32].

Nevertheless, while these findings may still support the role of perilesional and re-gional sampling, they also underscore the interpretive confusion at the heart of contemporary prostate biopsy: targeting too precisely may exaggerate grade by over-sampling the most aggressive microscopic focus, whereas targeting too narrowly, or with methods subject to inherent registration errors, may miss the true distribution of clinically significant disease [4]. In other words, the modern biopsy problem is no longer merely one of detection, but of reconciling spatial sensitivity and procedural targeting margins with biologically meaningful grading [21].

Therefore, the contemporary diagnostic challenge is not merely about blaming TBx for grade inflation, but rather reconciling the spatial sensitivity of MRI with biologically meaningful grading across the entire prostate [4,7,8,9].

## 3. Quantitative Evidence of Grade Discordance

Early analyses, such as the regional prostate network study by Kroon et al., highlighted the issue of grade overestimation by reporting a 15% downgrading rate in a cohort of 616 patients [9]. Notably, they found that the risk of downgrading was inversely correlated with the diameter of the MRI-detected lesion; smaller tumors (0–10 mm) carried an 18% risk of downgrading, whereas larger lesions (>20 mm) had a lower risk of 14%. According to these data, this risk of pathological downgrading is not uniform across all risk strata but is highly dependent on the initial biopsy Grade Group (GG). While the probability of downgrading remains remarkably low for GG 2 (approximately 2.7%), it becomes particularly pronounced for cases assigned a GG 4 following targeted biopsy, where a reported 61% of specimens were subsequently downgraded upon comprehensive analysis of the radical prostatectomy specimen.

A comprehensive meta-analysis by Weinstein et al., encompassing 6638 patients across 19 studies, revealed further insights into the differences between TBx, SBx, and combined biopsy approaches [8]. The authors found that both TBx alone and combined biopsies were significantly less likely to result in pathological upgrading at RP compared to SBx alone (upgrading rates of 27% for combined vs. 42% for SBx) [8]. However, this reduction in upgrading came at a specific cost: combined biopsies almost doubled the odds of downgrading at the time of RP (19.6% for combined vs. 11% for SBx) [8]. Similarly, Goel et al. demonstrated that SBx has a much higher likelihood of upgrading relative to TBx (Odds Ratio 2.47), confirming that TBx effectively targets the highest-grade disease but leaves room for potential grade overestimation [22].

It is advised, however, to separate pathological discordance from actual clinical overtreatment [33]. Downgrading a prostatectomy does not automatically imply that the radical treatment was inappropriate [4,8]. In a robust multicenter study of 1020 biopsy-naïve patients with Grade Group (GG) 2 on TBx, Baboudjian et al. reported an overall downgrading rate of 17% [4]. Yet, when defining strict “overtreatment” as cases that were downgraded to GG 1 or low-burden GG 2 (patients who could have been safely managed with AS), the actual overtreatment rate was only 2.7% [4]. Among 555 patients with GG 2 on TBx, merely 3.2% were downgraded to GG 1 at surgery [4].

Furthermore, Gaffney et al. evaluated 991 patients to assess the oncologic risk of discordant cores. They concluded that when the grade is discordant between systematic and MRI-targeted biopsies, the true oncologic risk (such as the risk of adverse pathology or biochemical recurrence) is intermediate between the two grades [34]. This evidence suggests that while TBx drives grade migration and increases the statistical rate of downgrading, the absolute risk of overtreatment is relatively low, and the high-grade elements detected by TBx still carry intermediate to high biological significance [4,34].

## 4. Pathological Mechanisms and the Relevance of Minor High-Grade Components

The fundamental mechanisms driving biopsy-to-prostatectomy grade discordance are deeply rooted in the grading protocols established by the International Society of Urological Pathology (ISUP) [29,35,36,37]. For a prostate biopsy, the Gleason score is calculated by adding the most prevalent pattern to the highest-grade pattern present, regardless of its volumetric percentage within the core [9,31,35,36]. Conversely, in a radical prostatectomy specimen, the score is derived by adding the most prevalent pattern to the second-most prevalent pattern, provided that the secondary pattern constitutes more than 5% of the total tumor volume [9,36]. Consequently, a targeted biopsy of a highly suspicious MRI focus may capture a concentrated pocket of Gleason pattern 4 [38]. If this pattern 4 does not meet the 5% volumetric threshold across the entire surgical specimen, the patient is formally “downgraded” [9,22].

However, the discussion of downgrading must better acknowledge the clinical relevance of minor high-grade components. The presence of a small-volume high-grade disease focus detected via TBx should not be summarily dismissed as a mere statistical artifact or clinically irrelevant over-precision [39]. Advanced histopathological features, such as a tertiary Gleason pattern 5, intraductal carcinoma (IDC), and invasive cribriform carcinoma, have been shown to be predictive of adverse oncologic outcomes, including biochemical recurrence and metastatic progression [6,29,35]. The 2019 ISUP consensus strongly advocates for the reporting of these architectures, as patients harboring these features have a biologically more aggressive disease phenotype compared to those with pure GG 1 or GG 2 without cribriform patterns [6,29]. Therefore, if TBx successfully identifies a minor focus of cribriform pattern 4 that prompts curative treatment, this should be viewed as successful targeted detection of an aggressive subclone, rather than a failure leading to overtreatment [29].

## 5. Implications for Risk Stratification and Focal Therapy

The precision paradox has profound implications for contemporary risk stratification and the application of Focal Therapy (FT) [40,41]. FT modalities (such as high-intensity focused ultrasound (HIFU), irreversible electroporation (IRE), and cryotherapy) aim to selectively ablate the “Index Lesion” while sparing the surrounding healthy prostatic tissue, neurovascular bundles, and urinary sphincter [32,41,42]. The success of FT appears to depend on the precise spatial and histological characterization provided by mpMRI and TBx [40,41,43].

If TBx overestimates the histological grade of a small index lesion, excellent candidates for organ-sparing conservative therapies might be erroneously redirected toward radical surgery or whole-gland radiotherapy [28]. Conversely, undersampling the perilesional “penumbra” can lead to incomplete ablation margins [28,31]. Research on FT margins indicates that incorporating intralesional heterogeneity and utilizing standardized 5–6 mm ablation margins around MRI-visible lesions could ensure the eradication of high-grade components [27].

Current clinical risk frameworks, such as the D’Amico classification or the NCCN guidelines, were largely developed and calibrated in the pre-MRI era based on systematic sampling [2,44,45]. Applying these historical nomograms directly to MRI-targeted data can generate a prognostic mismatch [34]. While it is reasonable to suggest that these models require updating, such recommendations must be phrased cautiously. Established risk frameworks are not entirely invalidated by mpMRI; rather, they require recalibration [46]. Future models should integrate focal grade data with spatial volume, PSA density (PSAD), and perilesional sampling outcomes to refine patient allocation without reflexively assuming that all TBx-upgraded cancers are overtly dangerous [7,24].

## 6. The Role of Biomarkers, Genomics, and Multi-Omics

To address the bidirectional tension of the precision paradox, the urological community is increasingly turning to molecular biology. Relying solely on morphological grading from biopsy cores is often insufficient to capture the true biological potential of a tumor [2,47]. Integrating genomic classifiers, tissue biomarkers, and multi-omics profiling could offer a robust mechanism to distinguish truly aggressive lesions from indolent ones that happen to feature a small focus of unfavourable architecture [48].

Validated tissue-based genomic classifiers, such as the Oncotype DX Genomic Prostate Score (GPS), the Prolaris Cell Cycle Progression (CCP) score, and the Decipher Genomic Classifier, have demonstrated significant clinical utility [2]. For example, the Decipher test, which evaluates 22 RNA features associated with cellular proliferation and androgen signaling, can accurately predict the risk of adverse pathology, biochemical recurrence, and metastasis independent of standard clinical variables [49]. Studies have shown that utilizing Decipher on biopsy cores can stratify patients with favorable intermediate-risk disease, helping to identify those who may safely continue AS despite a focal high-grade finding, versus those who harbor occult aggressive disease requiring immediate intervention [2,40,49]. Similarly, the Prolaris CCP score combines molecular risk with clinical features to provide a superior 10-year prostate cancer-specific mortality risk assessment, aiding in the appropriate selection of AS candidates [2,25].

Beyond transcriptomic panels, recent advances in multi-omics (integrating transcriptomics, proteomics, and metabolomics) provide deeper insights into tumor behavior [47,48]. For instance, comprehensive single-cell and chromatin accessibility analyses have identified specific metabolic regulators, such as Enolase 1 (ENO1) and Creatine Kinase B (CKB), which may play pivotal roles in PCa progression [47]. ENO1 upregulation is heavily associated with enhanced glycolysis, immunosuppression, and metastatic progression, whereas CKB expression correlates with a favorable prognosis and robust immune cell infiltration [47]. The integration of AI-driven multimodal models that combine deep learning of MRI suspicion levels, digital pathology, clinical data (age, PSAD), and genomic/metabolic profiles could significantly outperform traditional PSA-based or morphology-based strategies [48]. By using these multi-omics insights, clinicians can look beyond the focal biopsy grade, utilizing the tumor’s metabolic and genomic signature to mitigate the risks of both underdiagnosis and overtreatment [47].

## 7. Patient-Physician Counseling and Shared Decision Making

The nuances of the Precision Paradox necessitate a refined approach to patient-physician counseling [50]. The terminology surrounding “targeted” and “fusion” biopsy inherently implies absolute certainty, which can lead patients to assume that a high-grade finding in a single targeted core represents massive, life-threatening disease [50]. This can generate severe anxiety and prompt impulsive demands for radical treatment.

Physicians are advised to educate patients about the difference between a focal biopsy grade and the whole-gland disease burden. Counseling should explicitly frame the results within the context of the patient’s overall risk profile, explaining that while mpMRI is highly effective at finding the most aggressive cells, a small volume of pattern 4 disease within a tiny lesion might not preclude them from organ-sparing therapies.

To improve counseling, clinicians should transparently discuss all findings—including PSA density, the total number of positive systematic and targeted cores, and the PI-RADS score [7,50]. Highlighting the interplay between factors to help patients understand their personalized risk [7]. Furthermore, when molecular assays are utilized, the results should be clearly communicated to foster a shared decision-making process that aligns oncological safety with the preservation of quality of life and the reduction in treatment-associated morbidity [2,40,51,52].

## 8. Conclusions

The integration of mpMRI and targeted biopsy has been shown to improve the diagnosis of clinically significant prostate cancer. However, the resulting “Precision Paradox” requires clinicians to navigate the opposing tension between focal overestimation and spatial undersampling. While TBx increases the statistical rate of pathological downgrading at surgery compared to systematic biopsy, the absolute risk of true clinical overtreatment remains relatively low. Furthermore, minor components detected by TBx, such as cribriform architecture, carry genuine biological risk and should not be overlooked. To mitigate the risks of misclassification, a balanced diagnostic strategy is paramount. Combining targeted biopsies with perilesional or regional sampling offers a highly effective method to capture the true disease extent while minimizing the overdiagnosis of indolent cancers associated with random systematic sampling. Ultimately, the future of prostate cancer management lies in moving beyond isolated morphological grading. By systematically integrating MRI volumetrics, PSA density, multi-omics, and validated genomic classifiers into updated risk models, clinicians could achieve calibrated, personalized patient care that maximizes oncological efficacy while meticulously avoiding the harms of overtreatment.

## Data Availability

No new data were created or analyzed in this study.

## Abbreviations

The following abbreviations are used in this manuscript:

GG	Grade Group
ISUP	International Society of Urological Pathology
mpMRI	Multiparametric Magnetic Resonance Imaging
MRI	Magnetic Resonance Imaging
PCa	Prostate Cancer
PSA	Prostate-Specific Antigen
ROI	Region of Interest
RP	Radical Prostatectomy
SB	Systematic Biopsy
TB	Targeted Biopsy
AI	Artificial Intelligence
AS	Active Surveillance
CCP	Cell Cycle Progression
CKB	Creatine Kinase B
csPCa	Clinically Significant Prostate Cancer
EAU	European Association of Urology
ENO1	Enolase 1
FT	Focal Therapy
GPS	Genomic Prostate Score
HIFU	High-Intensity Focused Ultrasound
IDC	Intraductal Carcinoma
IRE	Irreversible Electroporation
NCCN	National Comprehensive Cancer Network
PI-RADS	Prostate Imaging Reporting and Data System
PSAD	PSA Density
SBx	Systematic Biopsy
TBx	Targeted Biopsies
TRUS	Transrectal Ultrasound
RNA	Ribonucleic Acid
